# Supplementary material for: MRI Study of Paraspinal Muscles in Patients with Amyotrophic Lateral Sclerosis (ALS)
Source: J Clin Med. 2020 Mar 28;9(4):934. doi: 10.3390/jcm9040934 (PMC7230865; doi:10.3390/jcm9040934)

Table S1. T1–weighted muscle MRI findings

A relative degree of severity was assigned to each muscle in each region, using the scoring system proposed by Mercuri (see Methods section).

HC=healthy control, IM=inflammatory myopathy, LR=lumbar radiculopathy

Paraspinal/pelvis: MF multifidus muscle, LD longissimus dorsi muscle, IC iliocostal muscle, P psoas muscle.

**Patient ID (years)**	**Paraspinal/pelvis**			
	**MF**	**LD**	**IC**	**P**
1 (29)	0	0	0	0
2 (52)	1	0	0	0
3 (39)	2a	1	1	0
4 (74)	1	1	2a	0
5 (65)	2a	2a	2a	0
6 (64)	0	0	0	0
7 (55)	2a	1	1	0
8 (76)	2b	2b	2b	0
9 (73)	2a	2a	2a	1
10 (64)	2a	1	2a	0
11 (56)	2a	1	2a	0
12 (69)	2a	1	1	0
13 (63)	2a	1	1	0
14 (79)	1	1	1	0

**HC ID (years)**	**Paraspinal/pelvis**			
	**MF**	**LD**	**IC**	**P**
1 (56)	1	1	1	0
2 (69)	1	1	1	0
3 (72)	2a	2a	2a	0
4 (67)	1	1	1	0
5 (48)	1	2a	2a	0
6 (76)	1	1	1	0
7 (75)	1	1	2a	1
8 (61)	1	1	1	0
9 (76)	2a	2a	2a	1
10 (63)	2a	1	2a	0

11 (67)	2a	2a	2a	1

IM ID (years)	Paraspinal/pelvis			
	MF	LD	IC	P
1 (29)	0	0	0	0
2 (62)	1	1	1	0
3 (60)	2b	1	2a	1
4 (24)	1	1	1	0
5 (49)	2a	2a	2a	1
6 (39)	2a	1	2a	0
7 (42)	2a	2a	2a	0
8 (49)	1	1	2a	0
9 (53)	3	3	3	0
10 (53)	2a	2a	2a	0

LR ID (years)	Paraspinal/pelvis			
	MF	LD	IC	P
1 (57)	2a	1	2a	0
2 (51)	1	0	1	0
3 (44)	2a	1	1	0
4 (63)	2a	2a	2a	1
5 (71)	2a	2a	2a	1
6 (66)	2b	2a	2a	1
7 (63)	2a	1	1	0
8 (64)	2b	2a	2a	1
9 (68)	1	1	2a	1
10 (57)	1	1	1	0
11 (64)	1	1	2a	0
12 (51)	1	1	2a	0
13 (60)	1	1	2a	0
14 (70)	2b	2a	2b	1
15 (75)	2b	2b	2b	1
16 (64)	2a	1	1	1
17 (67)	2b	2a	2a	0
18 (62)	1	1	2a	0
19 (50)	2a	1	2a	1

Figure S1. MRI scans of patient with myopathy (a, c patient – #1) and patient with radiculopathy (b, d – patient #12): paraspinal axial (a – patient #1, b – patient #12), and coronal T1 (c – patient #1, d – patient #12) images show no fatty replacement in the patient with myopathy and a mild degree of fatty replacement in the patient with radiculopathy.

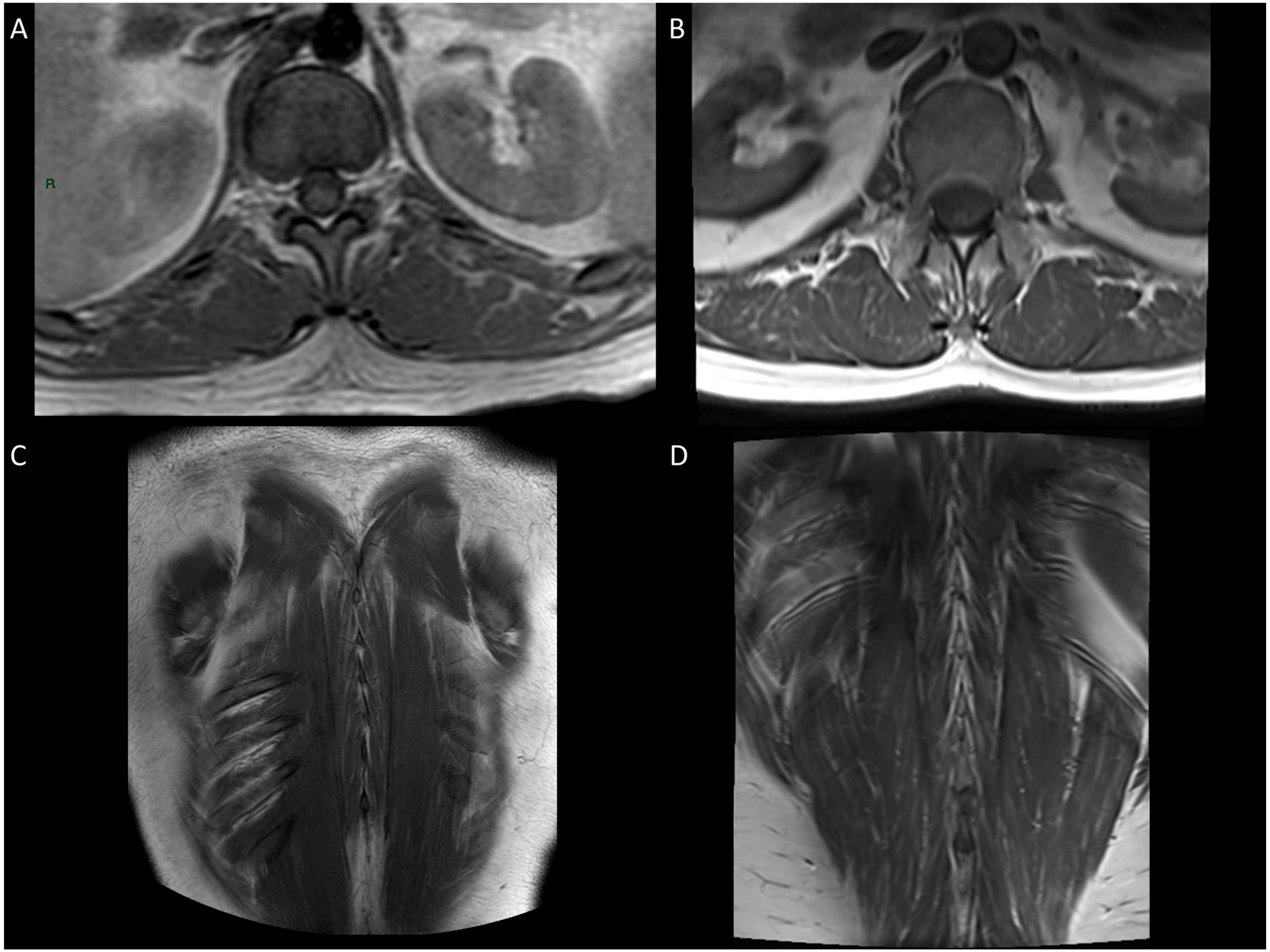

Supplement: Supplementary file 1 [file jcm-09-00934-s001.pdf]
